# An Evaluation and Ranking of Children’s Hospital Websites in the United States

**DOI:** 10.2196/jmir.5799

**Published:** 2016-08-22

**Authors:** Timothy R Huerta, Daniel M Walker, Eric W Ford

**Affiliations:** ^1^ The Ohio State University, College of Medicine Department of Family Medicine Columbus, OH United States; ^2^ Johns Hopkins Bloomberg School of Public Health Department of Health Policy and Management Baltimore, MD United States

**Keywords:** pediatric hospital, Internet, social media, patient participation, patient education

## Abstract

**Background:**

Children’s hospitals are faced with the rising need for technological innovation. Their prospective health care consumers, who increasingly depend on the Web and social media for communication and consumer engagement, drive this need. As patients and family members navigate the Web presence of hospitals, it is important for these specialized organizations to present themselves and their services efficiently.

**Objective:**

The purpose of this study was to evaluate the website content of children’s hospitals in order to identify opportunities to improve website design and create benchmarks to judge improvement.

**Methods:**

All websites associated with a children’s hospital were identified using a census list of all children’s hospitals in the United States. In March of 2014, each website and its social media were evaluated using a Web crawler that provided a 5-dimensional assessment that included website accessibility, marketing, content, technology, and usability. The 5-dimensional assessment was scored on a scale ranging from 0 to 10 with positive findings rated higher on the scale. Websites were ranked by individual dimensions as well as according to their average ranking across all dimensions.

**Results:**

Mean scores of 153 websites ranged from 5.05 to 8.23 across all 5 dimensions. Results revealed that no website scored a perfect 10 on any dimension and that room exists for meaningful improvement.

**Conclusions:**

Study findings allow for the establishment of baseline benchmarks for tracking future website and social media improvements and display the need for enhanced Web-based consumer engagement for children’s hospitals.

## Introduction

More and more, patients seek health-related information from Internet sources [1]. The trend is particularly strong among teens and young adults, as these demographics show the highest rate of Internet usage both in general and specifically for health information [2-4]. The increased use of Web-based technology to seek health information provides opportunities for health care organizations to create innovative platforms to educate and engage their consumers. Despite the promise of greater information availability, consumer-focused Web interfaces have not advanced as quickly in the health care industry as in other sectors [5].

By virtue of the fact that children’s hospitals serve younger demographics, they may be particularly well positioned to capitalize from an effective Web presence to attract new patients and provide high-fidelity information [6-8]. For example, a study of diabetic children’s parents found that they have significant desire for a Web-based platform to augment clinical services. However, participants expressed concerns, including whether the websites’ information was current and their own abilities to discern whether information was credible [9,10]. Therefore, information sources may not be reliable and children may lack the health literacy needed to assess content credibility [11].

Children’s hospital websites are well positioned to explore concerns about consumer-targeted information’s reliability and relevance [12]. In addition, they can act as a portal for patient-specific health-related information and serve as a comprehensive source to engage patients with the health care system early in their lives. Thus, it is important to understand the current state of the art in children’s hospital websites for consumers, providers, and health information content creators. The purpose of this short paper is to identify the degree to which children’s hospital websites adhere to Internet industry standards for usability [13]. Using Internet industry standards provides a reference point against which to assess the children’s hospital websites and enables the establishment of benchmarks that can help children’s hospitals identify areas for improvement as they aim to optimize the use of Web-based technology to engage their patients.

## Methods

A list of 157 US children’s hospitals with unique website domains linked to the named institutions was compiled from the Children’s Hospital Association’s membership in 2014. For websites that were part of a general hospital domain, only the sections of the website pertaining specifically to the children’s hospital were included in the analysis. Website analyses were simultaneously performed in March of 2014.

Procedures for the website analytic tool paralleled previous health care website assessments [14,15]. The automated tool, termed the “Web crawler,” was created to navigate and assess websites’ functionalities. The Web crawler assessed 24 metrics about each website (eg, broken links, content readability). A complete list of these indices and their description is provided in Multimedia Appendix 1. Different subsets of the 24 metrics were weighted and combined to create 5 indices: accessibility, content, marketing, technology, and usability (see Multimedia Appendix 1 for weighting) [15,16].

The analysis algorithm begins with the Web crawler developing a map of the website from its front or “top” page down through all pages associated with the children’s hospital Web domain to a maximum of 1000 pages’ breadth. Once the sampled pages for each hospital are mapped, the systematic tool surveys the pages for each component that comprises each of the 5 dimensions. The dimension of accessibility refers to the ability of those with low levels of computer literacy to access and navigate the hospital’s Web presence. The marketing dimension is composed of items such as a website’s ability to be found through search engines, examining the relevance of descriptions to the links provided, and ranking of the website in performed searches. Content dimensionality refers to items such as the grammar of website text, the frequency of information updates, material relevancy, and readability metrics. The technology dimension is scored by how quickly the website downloads, the quality of the programming code, the website’s infrastructure, and how it performs. The fifth dimension of website assessment is usability. The usability dimension provides an overall assessment of website quality and is formed as a composite of the metrics used in the other indices, rather than a composite of the other indices themselves. Please see Huerta et al [15] for a more detailed breakdown and itemization of the process.

Websites were assigned a score for each of the 5 dimensions, ranging from 0 to 10, with a comparatively better rating reflected with a higher score. After dimension scores were determined, hospitals were assigned a rank for their score in each dimension. An overall rank was determined by averaging the ranks of all 5 dimensions. For the overall rank, we used the average rank from the 5 dimensions rather than the average score to keep the relative nature of the scores intact in our overall ranking.

## Results

During the analytic process, technical issues prevented running the Web crawler on 4 of the 157 sites, leaving them unanalyzed and prompting their removal from the test sample. These technical issues were likely due to tools on the website servers that prevented “pings” against their website. However, the exact cause of these technical issues remains unknown, as they arose from issues with the server rather than the Web crawler. Of the 5 dimensions evaluating Web domains for the included 153 children’s hospitals in the United States, the dimension of content had the highest average hospital website score at 8.23 on a scale from 1 to 10, as well as the largest range. The accessibility dimension had the lowest average assessment value at 5.05. Mean scores for the other dimensions ranged from 5.36 to 6.7. Summary statistics for the 5 dimensions are presented in Table 1.

**Table 1:** Children’s hospital website summary statistics for each dimension assessed by the Web crawler.

Dimension	Mean	Standard deviation	Minimum	Maximum
Accessibility	5.05	1.29	0.7	7.4
Content	8.23	1.15	1.2	9.7
Marketing	6.73	1.17	1.7	8.8
Technology	5.36	1.87	0	7.7
Usability	6.13	1.39	1.7	8.1

The overall rankings, and the rankings and scores for each dimension, for the top 100 overall rated children’s hospital websites are reported in Multimedia Appendix 2. On the basis of rankings, the top overall performing children’s hospital website was the Children’s Hospital of Philadelphia. This hospital had the top performing website for both marketing and technology, the 2nd highest score for usability, the 6th highest for content, and the 10th best for accessibility.

## Discussion

### Principal Findings

This study provides a comprehensive and thorough assessment of children’s hospital websites. The growing demand for technological innovation combined with an increasing reliance on Web-based information among the age demographic most heavily represented in children’s hospitals is essential for these hospitals to bear in mind. A well-designed website that adheres to national standards and demonstrated best practices is of greater importance for issues related to care access, as infirm children and their families may be ill-equipped to overcome barriers to receiving care [17,18].

The results of this study can help children’s hospitals to assess their own Web presence relative to their peers, providing a basis to improve Web services. Hospitals ranked lower on the list within each domain, or overall, can look to the top performers for guidance on how best to design their websites. The difference between low and high rankings may not require complete redesign, but rather may entail straightforward solutions, such as fixing broken links, correcting misspelled items, adding Twitter links, or offering Spanish-language services—all common yet easily fixable pitfalls. Moreover, the high-ranking hospitals appear to use their websites to orient, engage, and inform their visitors, rather than solely to provide visitors with basic information. These high-ranking hospitals view their websites as critical to an ongoing relationship with patients. The lesson for the lower-ranked hospitals is that a website can enlist features that promote health and wellness and also engage patients in their care. For example, websites can host Web-based interventions [19] or offer useful patient education materials and disease management materials [20].

### Comparison With Prior Work

The authors are unaware of any similar previous studies done on children’s hospitals. As a result, no prior work exists that provides a useful comparison for the findings in this study. However, earlier in 2014, a study with similar methodology was performed on 2407 acute care general hospital website domains [15]. For a point of relative comparison, a side-by-side comparison of the two studies is presented in Table 2. The study found a mean usability score of 5.16 for US hospitals, which is comparably lower to the score found in the usability dimension in our study. This difference may stem from a higher likelihood of children’s hospitals to be connected with large, academic facilities. Both large and academic medical centers may have more resources available for Web design, thereby producing higher scores for children’s hospitals on average. Children’s hospitals could also recognize the opportunity to use their websites to educate and engage patients in their target demographic relatively more than general hospitals.

**Table 2:** Comparison of Web crawler analytic scores for websites of US acute care general hospitals [15] and children’s hospitals.

Dimension	US hospitals^a^ Mean (SE^b^)	Children’s hospitals Mean (SE)
Accessibility	5.08 (0.05)	5.05 (0.10)
Content	6.49 (0.02)	8.23 (0.09)
Marketing	5.03 (0.03)	6.73 (0.09)
Technology	4.43 (0.04)	5.36 (0.15)
Usability	5.16 (0.03)	6.13 (0.11)

^a^Acute care general hospitals.

^b^SE: standard error.

### Limitations

Several important limitations affect this study. Previous studies of this nature have excluded facilities associated with an education top-level domain (.edu) [14,15]; however, this domain is prevalent enough in children’s hospitals to warrant inclusion. As a result, there is a risk of inclusion of academic departments or other such pages unrelated to patient care or the hospital that could skew assessment. It should be noted that the hospitals are generally confined to a smaller subsection of a .edu domain, decreasing the risk of an unrelated academic side being evaluated.

The second limitation influencing this study relates to website size. A website can have several pages associated with it or can be more limited in scope. The Web crawler does not directly adjust for this size component; this results in websites with a few Web pages and narrowly focused high-quality content potentially being rated the same as websites with numerous pages and equivalently high-quality content that is broader in scope. Thus, interpreting a single overall score, although convenient, may negate the diversity of hospitals’ Web design.

Third, our study assessed websites in the spring of 2014. The Internet is a rapidly evolving landscape, websites are continuously improving, and the best demonstrated practices are evolving. As a result, our findings may not represent the current status of each hospital’s websites. Nonetheless, we view our assessment of children’s hospital website design as establishing a baseline with which future comparisons can be made and against which progress can be measured.

Finally, our use of a Web crawler as the key measurement tool in this study biases our results toward items discernible by the technology. More nuanced website features may be indiscernible by the Web crawler: for example, the Web crawler may miss website layout issues that influence usability. Alternatively, the use of an established Web crawler does ensure high levels of validity and reliability to our assessment. Ultimately, our study achieves its aim to evaluate the websites to better understand the development of this infrastructure but does not evaluate end-user satisfaction and engagement that may lead to website use and impact. Understanding how specifically consumers are interacting with technology is a ripe area for future research.

### Conclusions

This analysis presents a systematic assessment of children’s hospital Web and social media presence. Giving consideration to the increasing societal expectations for technology, in particular those of the younger demographic, the number of poorly performing facilities across all the calculated scores is a cause for concern in the near term. The social media and Web presence of children’s hospitals may represent the first contact patients make with an organization. This initial contact must engage the patient, or else they may explore alternative treatment options or not recognize care services available to them. This consequence may not only have a negative impact on the hospital, particularly in more competitive markets, but also lead to suboptimal care processes and outcomes for the patient. Improving website design, complying with Internet industry standards, and optimizing search engine performance can maximize the potential power of the Internet to engage and inform patients.

## Appendix

Multimedia Appendix 1 Scale components and weightings.

Multimedia Appendix 2 Ranking of the top 100 children’s hospital websites for each dimension and an average ranking across dimensions.
