# Supplementary material for: An Evaluation and Ranking of Children’s Hospital Websites in the United States
Source: J Med Internet Res. 2016 Aug 22;18(8):e228. doi: 10.2196/jmir.5799 (PMC5011553; doi:10.2196/jmir.5799)
Supplement: Multimedia Appendix 1 [file jmir_v18i8e228_app1.pdf]

Table 1: Scale components and weightings

Assessment components	Description	Accessibility	Content	Marketing	Technical	Usability	all categoriesUsage across
Alternative text	Assesses whether alternative text accompanies images	15%		4%	4%	5%	6%
Amount of content	Measures the number of pages with a reasonable amount of text	8%	33%	9%		5%	9%
Analytics	Determines whether website implements software packages to track new and recurring visitors, which web pages readers visit and for how long, etc.			4%	4%	3%	3%
Broken links	Assesses whether the site contains links to web addresses that do not exist or return an error	8%		1%	8%	5%	4%
Domain age	Identifies the original registration date of the organization's domain to determine age. This is a component of SEO systems			4%			1%
Error pages	Checks for error messages and faults. Such errors are strongly suggestive of technical problems in the website			4%	8%	3%	3%
Facebook	Checks whether the website has a Facebook page, Facebook group, or personal Facebook profile and scores that site by the number of likes published by Facebook			8%		4%	3%
Headings	Assesses whether headings are used effectively throughout the site to improve search engine placement, accessibility, and usability	8%		4%	8%	5%	5%
Incoming links	Measures the number of links from major search engines, including Yahoo! and Google			9%		5%	4%
Link states	Assesses the use of cascading style sheets (CSS) - the industry standard for page	15%			8%	4%	5%

	design, font sizing, and style						
Missing files	Assessed the absence to referred files on the page, resulting in failed page construction				8%	3%	2%
Open Graph	Open Graph tags are the emerging standard to facilitate sharing of web content on social media			4%	6%	4%	3%
Popularity	Calculates the relative Alexa ranking of popularity compared to other websites and whether the ranking is rising or falling; Alexa combines the viewing history of many web browsers using particular browser toolbars			9%		6%	4%
Printability	Determines whether a web page is designed to be printed and whether specific CSS style sheets are designed for printing				8%	5%	3%
Readability	Assesses the comprehensibility and quality of site content using the Flesch-Kincaid Reading Ease and Gunning Fog Index scales	8%	33%	2%		5%	7%
Redirections	Assesses the ability to move backward and forward across pages using browser buttons	6%			6%	1%	2%
Search engine results	Determines how text from a web page appears in Google search results	4%	13%	7%	4%	5%	6%
Social interest	Checks the amount of social interest that individual pages within the website have. Heavily reliant on Facebook Likes and Twitter for its calculation.			11%		5%	5%
Speed	Assesses the use of files in the website— specifically, how large the files are, how long they take to download, and whether any are missing	6%		3%	15%	10%	7%
Spelling	Determines whether the words on a page are spelled correctly		20%				2%
Stylesheets	Determines whether and how effectively CSS is used throughout the site, such as avoiding embedded style	8%		1%	6%	5%	4%

	sheets and tables for website positioning						
Twitter	Determines whether the website has a Twitter account and how often the account is referred to			7%		3%	3%
URL format	Assesses the use of natural language to improve the readability of the address for each page	7%		7%	4%	4%	5%
W3C compliance	Assesses whether the website implements best practices and meets specifications for site development according to the W3C, a consortium and standards body of web technologies	8%			8%	5%	4%

Note: Percentages do not add to 100 for rounding reasons.
