# Supplementary material for: An Evaluation and Ranking of Children’s Hospital Websites in the United States
Source: J Med Internet Res. 2016 Aug 22;18(8):e228. doi: 10.2196/jmir.5799 (PMC5011553; doi:10.2196/jmir.5799)
Supplement: Multimedia Appendix 2 [file jmir_v18i8e228_app2.pdf]

Multimedia Appendix 2. Ranking of the top 100 children's hospital websites for each dimension and an average ranking across dimensions.

Children's hospital name	Accessibility		Content		Marketing		Technology		Usability		Overall	
	Score	Rank	Score	Rank	Score	Rank	Score	Rank	Score	Rank	Average Rank	Over-all Rank
The Children's Hospital of Philadelphia	6.5	10	9.1	6	8.8	1	8.1	1	7.6	2	4	1
Nationwide Children's Hospital	6.5	10	9	10	8.5	2	8	2	7.1	27	10.2	2
Children's Hospital of Wisconsin	6.2	18	8.9	17	8	12	7.8	4	7.6	2	10.6	3
Doernbecher Children's Hospital at the Oregon Health and Science University	6.3	16	8.9	17	8	12	7.6	7	7.3	15	13.4	4
Cincinnati Children's Hospital Medical Center	6	28	9	10	8.3	4	7.6	7	7.2	20	13.8	5
Children's Hospital Colorado	5.9	36	8.8	21	8.1	8	7.7	6	7.4	9	16	6
Mattel Children's Hospital University of California at Los Angeles	6.7	5	9.2	2	7.7	29	7.8	4	6.8	40	16	6
The Children's Hospital at Oklahoma University Medical Center	5.9	36	8.8	21	8.1	8	7.5	11	7.3	15	18.2	8
St. Jude Children's Research Hospital	5.8	48	9	10	8.4	3	7.9	3	7	31	19	9
The Children's Medical Center of Dayton	5.8	48	9.2	2	8	12	7.5	11	7	31	20.8	10
Children's Hospital of Pittsburgh of University of Pittsburgh Medical Center	5.5	71	9	10	8.2	6	7.6	7	7.2	20	22.8	11
University of Michigan C.S. Mott Children's and Von Voigtlander Women's Hospital	6	28	8.7	36	7.6	37	7.5	11	7.4	9	24.2	12
Children's Memorial Hermann Hospital	6.8	3	8.9	17	7.9	16	7.2	32	6.3	57	25	13

Children's hospital name	Accessibility		Content		Marketing		Technology		Usability		Overall	
	Score	Rank	Score	Rank	Score	Rank	Score	Rank	Score	Rank	Average Rank	Over-all Rank
Phoenix Children's Hospital	6	28	8.8	21	7.9	16	7.4	15	6.5	48	25.6	14
Blank Children's Hospital	5.9	36	9.1	6	7.6	37	7.3	22	7	31	26.4	15
Lucile Packard Children's Hospital at Stanford	6.1	23	8.8	21	7.2	59	7.3	22	7.4	9	26.8	16
Children's Hospital at Dartmouth Dartmouth-Hitchcock Medical Center	6.8	3	9.1	6	6.7	82	7.1	38	7.5	6	27	17
Miami Children's Hospital	6.2	18	9	10	8.1	8	7.1	38	5.9	69	28.6	18
Children's Medical Center Dallas	5.9	36	8.8	21	8.3	4	7.3	22	6	64	29.4	19
Yale New Haven Children's Hospital	5.9	36	8.7	36	7.6	37	7.3	22	7.1	27	31.6	20
University of Virginia Children's Hospital	6.6	7	8.8	21	7.8	20	7	48	6	64	32	21
University of California Davis Children's Hospital	5.9	36	8.5	66	7.7	29	7.4	15	7.2	20	33.2	22
Dell Children's Medical Center of Central Texas	6.1	23	8.5	66	7.2	59	7.4	15	7.4	9	34.4	23
University of Iowa Children's Hospital	5.5	71	8.6	51	7.8	20	7.4	15	7.3	15	34.4	23
The Mount Sinai Kravis Children's Hospital	6.6	7	8	128	7.7	29	7.5	11	7.7	1	35.2	25
Janet Weis Children's Hospital at Geisinger Medical Center	6.1	23	8.7	36	7.8	20	7.1	38	6	64	36.2	26
Children's Healthcare of Atlanta	5.5	71	8.8	21	8.2	6	7.3	22	6	64	36.8	27
Akron Children's Hospital	5.7	58	8.8	21	7.8	20	7.2	32	6.2	60	38.2	28
Diamond Children's Medical Center	5.9	36	8.5	66	7.4	49	7.3	22	7.2	20	38.6	29
Rady Children's Hospital - San Diego	5.8	48	8.5	66	7.6	37	7.4	15	7.1	27	38.6	29
American Family Children's Hospital	5.5	71	8.5	66	7.7	29	7.4	15	7.3	15	39.2	31

Children’s hospital name	Accessibility		Content		Marketing		Technology		Usability		Overall	
	Score	Rank	Score	Rank	Score	Rank	Score	Rank	Score	Rank	Average Rank	Over-all Rank
Hurley Children's Hospital	5.9	36	9.2	2	7.8	20	6.9	53	5.4	88	39.8	32
Hasbro Children's Hospital at Rhode Island Hospital	6.4	12	8.4	82	7.1	65	7.2	32	7.4	9	40	33
University of Minnesota Amplatz Children's Hospital	5.6	66	8.7	36	7.5	46	7.3	22	7	31	40.2	34
Seattle Children's	3.8	120	9.2	2	8.1	8	7.3	22	6.3	57	41.8	35
Gillette Children's Specialty Healthcare	5.9	36	8.5	66	7.7	29	7.2	32	6.4	53	43.2	36
Cleveland Clinic Children's Hospital for Rehabilitation	5	96	8.6	51	7.8	20	7.3	22	7	31	44	37
All Children's Hospital	4.7	105	8.8	21	7.4	49	7.3	22	7.1	27	44.8	38
Boston Children's Hospital	6	28	8.8	21	7.7	29	6.7	67	5.7	79	44.8	38
University of Maryland Children's Hospital	3.8	120	9	10	7.9	16	7.1	38	6.7	42	45.2	40
Cleveland Clinic Children's Hospital	4.6	108	8.6	51	7.8	20	7.4	15	6.9	37	46.2	41
University Hospitals Rainbow Babies and Children's Hospital	6.7	5	8.3	98	7.4	49	7.2	32	6.5	48	46.4	42
The Children's Institute	6	28	8.7	36	7.1	65	6.9	53	6.3	57	47.8	43
Greenville Health System Children's Hospital	7.4	1	8.1	122	6.9	75	7.1	38	7.5	6	48.4	44
Mt. Washington Pediatric Hospital, Inc	6.1	23	8.4	82	7.4	49	7	48	6.5	48	50	45
Children's National Medical Center	5.6	66	8.6	51	7.9	16	7.1	38	5.7	79	50	45
Miller Children's Hospital Long Beach	6.1	23	8.7	36	7.1	65	6.8	61	5.9	69	50.8	47
Golisano Children's Hospital at The University of Rochester Medical Center	5.8	48	8.3	98	7.7	29	7.1	38	6.7	42	51	48
Sutter Children's Center, Sacramento	6	28	8.2	109	6.7	82	7.1	38	7.6	2	51.8	49

Children's hospital name	Accessibility		Content		Marketing		Technology		Usability		Overall	
	Score	Rank	Score	Rank	Score	Rank	Score	Rank	Score	Rank	Average Rank	Over-all Rank
Brenner Children's Hospital, Wake Forest Baptist Health	5.5	71	8.9	17	6.7	82	6.8	61	6.9	37	53.6	50
Franciscan Hospital for Children	5.8	48	8.1	122	7.1	65	7.2	32	7.6	2	53.8	51
Children's Hospital and Research Center at Oakland	6.2	18	8.4	82	7.3	54	6.9	53	5.9	69	55.2	52
Upstate Golisano Children's Hospital	6.2	18	8.5	66	7.2	59	6.8	61	5.8	74	55.6	53
St. Christopher's Hospital for Children	5.8	48	8.7	36	7.6	37	6.7	67	5.3	92	56	54
East Tennessee Children's Hospital	5.7	58	8.4	82	7.7	29	7	48	6	64	56.2	55
The Goryeb Children's Hospital	7	2	8.2	109	6.4	100	6.9	53	7.2	20	56.8	56
Kennedy Krieger Institute	5.8	48	8.3	98	7.3	54	7.1	38	6.5	48	57.2	57
Primary Children's Medical Center	5.3	87	8.6	51	7.3	54	6.9	53	6.6	46	58.2	58
Women and Children's Hospital of Buffalo	5.7	58	8.6	51	6.9	75	6.8	61	6.6	46	58.2	58
Arkansas Children's Hospital	4.8	100	9.1	6	7.8	20	6.8	61	4.9	106	58.6	60
Cardon Children's Medical Center	5.2	92	8.7	36	7.3	54	6.9	53	6.2	60	59	61
Children's Hospital of Richmond at Virginia Commonwealth University	5.8	48	8.2	109	6.9	75	6.9	53	7.2	20	61	62
Cook Children's Medical Center	3.9	118	9	10	7.5	46	7	48	5.5	85	61.4	63
Mission Children's Hospital at Mission Health	5.7	58	8.7	36	6.7	82	6.6	74	6.2	60	62	64
Shriners Hospitals for Children - all sites	4.7	105	8.4	82	7.3	54	7.1	38	6.9	37	63.2	65
New York-Presbyterian, Morgan Stanley Children's Hospital	4.8	100	8.7	36	6.1	115	6.9	53	7.3	15	63.8	66
The Joseph M. Sanzari Children's	6.3	16	8.3	98	6.5	97	6.7	67	6.7	42	64	67

Children's hospital name	Accessibility		Content		Marketing		Technology		Usability		Overall	
	Score	Rank	Score	Rank	Score	Rank	Score	Rank	Score	Rank	Average Rank	Over-all Rank
Hospital Hackensack University Medical Center												
Walt Disney Pavilion at Florida Hospital for Children	5.7	58	8.7	36	7.1	65	6.5	77	5.2	94	66	68
Duke Children's Hospital and Health Center	5.3	87	8.3	98	7.2	59	7	48	6.8	40	66.4	69
MassGeneral Hospital for Children Massachusetts General Hospital	5.5	71	8.8	21	7.8	20	4.7	130	5.2	94	67.2	70
Kosair Children's Hospital	5.8	48	8.6	51	7	70	6.3	83	5.2	94	69.2	71
University of California at San Francisco Benioff Children's Hospital	5.5	71	8.6	51	7.2	59	6.7	67	5.1	99	69.4	72
Johns Hopkins Children's Center	6.4	12	8.6	51	7.4	49	4.6	132	5	103	69.4	72
St Louis Children's Hospital	4.4	112	8.8	21	7.6	37	6.7	67	4.6	111	69.6	74
St. Joseph's Children's Hospital	5.8	48	8.5	66	6.3	108	6.5	77	6.4	53	70.4	75
MultiCare Mary Bridge Children's Hospital and Health Center	5.9	36	8.1	122	7.6	37	6.7	67	5.2	94	71.2	76
Children's Hospital of Illinois at OSF Saint Francis Medical Center	6	28	8.8	21	7	70	4.2	137	4.5	113	73.8	77
Riley Hospital for Children at Indiana University Health	5.5	71	9.7	1	7.6	37	4.4	134	3.9	127	74	78
K. Hovnanian Children's Hospital at Jersey Shore University Medical Center	5.4	81	8.7	36	6.4	100	6.4	81	5.8	74	74.4	79
Nemours Children's Hospital	5.7	58	8.7	36	8	12	4.3	135	3.2	132	74.6	80
Sacred Heart Children's Hospital	5.5	71	8.1	122	6.4	100	6.8	61	7.2	20	74.8	81

**Children's hospital name**	**Accessibility**		**Content**		**Marketing**		**Technology**		**Usability**		**Overall**	
	Score	Rank	Score	Rank	Score	Rank	Score	Rank	Score	Rank	Average Rank	Over-all Rank
Blythedale Children's Hospital	5.9	36	8.2	109	7.2	59	6.5	77	5.2	94	75	82
McLane Children's Scott and White	5.5	71	8.5	66	6.8	81	6.3	83	5.5	85	77.2	83
Helen DeVos Children's Hospital	5.4	81	8.6	51	6.6	91	6.3	83	5.6	81	77.4	84
Children's Mercy Hospital	4.5	110	8.5	66	6.7	82	6.6	74	6.1	63	79	85
West Virginia University Children's Hospital	5.6	66	8.3	98	5.9	126	6.5	77	7	31	79.6	86
Ann and Robert H. Lurie Children's Hospital of Chicago	4.9	99	8.6	51	7	70	6.1	96	5.4	88	80.8	87
Children's Hospital of Michigan	5.4	81	8.2	109	7.6	37	6.6	74	5	103	80.8	87
Loma Linda University Children's Hospital	5.2	92	8.5	66	6.6	91	6.4	81	5.6	81	82.2	89
Texas Children's Hospital	2.7	146	8.4	82	7	70	6.7	67	6.5	48	82.6	90
The Bristol-Myers Squibb Children's Hospital at Robert Wood Johnson University Hospital	5.3	87	8.4	82	6.4	100	6.3	83	5.8	74	85.2	91
North Carolina Children's Hospital University of North Carolina Hospitals	6.2	18	7.9	132	6.6	91	6.2	92	5.1	99	86.4	92
Memorial Children's Hospital	6	28	4.6	149	5.1	142	5.7	110	7.4	9	87.6	93
El Paso Children's Hospital	5.9	36	8.2	109	6.6	91	6	99	5	103	87.6	93
Arnold Palmer Hospital for Children	4.8	100	8.6	51	7	70	6.1	96	4.2	121	87.6	93
La Rabida Children's Hospital	6.6	7	8.2	109	6.4	100	6.1	96	2.7	135	89.4	96
Beaumont Children's Hospital	3.5	133	8.8	21	6.7	82	5.9	106	4.8	108	90	97
Hoops Family Children's Hospital	6.4	12	7.9	132	5.7	129	6	99	5.6	81	90.6	98
Le Bonheur Children's Hospital	2.7	146	8.4	82	7.5	46	6.3	83	5.1	99	91.2	99
The Children's Hospital at Saint Francis	3.9	118	8.6	51	5.4	137	6	99	6.4	53	91.6	100
